# Intramuscular Injection of miR-1 Reduces Insulin Resistance in Obese Mice

**DOI:** 10.3389/fphys.2021.676265

**Published:** 2021-07-06

**Authors:** Alice C. Rodrigues, Alexandre R. Spagnol, Flávia de Toledo Frias, Mariana de Mendonça, Hygor N. Araújo, Dimitrius Guimarães, William J. Silva, Anaysa Paola Bolin, Gilson Masahiro Murata, Leonardo Silveira

**Affiliations:** ^1^Department of Pharmacology, Instituto de Ciencias Biomedicas, Universidade de São Paulo, São Paulo, Brazil; ^2^Obesity and Comorbidities Research Center (OCRC), Campinas, Brazil; ^3^Department of Structural and Functional Biology, Institute of Biology, University of Campinas (UNICAMP), Campinas, Brazil; ^4^Department of Anatomy, Instituto de Ciencias Biomedicas, Universidade de São Paulo, São Paulo, Brazil; ^5^Department of Medical Clinics, Faculty of Medicine, University of São Paulo, São Paulo, Brazil

**Keywords:** skeletal muscle, mitochondrial dysfunction, high-fat diet, obesity, microRNA

## Abstract

The role of microRNAs in metabolic diseases has been recognized and modulation of them could be a promising strategy to treat obesity and obesity-related diseases. The major purpose of this study was to test the hypothesis that intramuscular miR-1 precursor replacement therapy could improve metabolic parameters of mice fed a high-fat diet. To this end, we first injected miR-1 precursor intramuscularly in high-fat diet-fed mice and evaluated glucose tolerance, insulin sensitivity, and adiposity. miR-1-treated mice did not lose weight but had improved insulin sensitivity measured by insulin tolerance test. Next, using an *in vitro* model of insulin resistance by treating C2C12 cells with palmitic acid (PA), we overexpressed miR-1 and measured p-Akt content and the transcription levels of a protein related to fatty acid oxidation. We found that miR-1 could not restore insulin sensitivity in C2C12 cells, as indicated by p-Akt levels and that miR-1 increased expression of *Pgc1a* and *Cpt1b* in PA-treated cells, suggesting a possible role of miR-1 in mitochondrial respiration. Finally, we analyzed mitochondrial oxygen consumption in primary skeletal muscle cells treated with PA and transfected with or without miR-1 mimic. PA-treated cells showed reduced basal respiration, oxygen consumption rate-linked ATP production, maximal and spare capacity, and miR-1 overexpression could prevent impairments in mitochondrial respiration. Our data suggest a role of miR-1 in systemic insulin sensitivity and a new function of miR-1 in regulating mitochondrial respiration in skeletal muscle.

## Introduction

Obesity, an abnormal or excessive fat accumulation that presents a risk to health, is strongly associated with type 2 diabetes mellitus (T2DM) (Boles et al., 2017). Being obese increases the risk of developing T2DM by at least six times, regardless of genetic predisposition to the disease (Schnurr et al., 2020). Intramyocellular lipid accumulation, resulting from enhanced adipose tissue lipolysis and impaired fatty acid beta-oxidation in skeletal muscle, induces insulin resistance, a major defect involved in the development of T2DM (Sergi et al., 2019). Mitochondrial reduced function in muscle has been associated with this metabolic inflexibility observed in T2DM patients (Phielix et al., 2008). Therefore, enhancing mitochondrial function in skeletal muscle may be a strategy to improve insulin sensitivity.

MicroRNAs (miRNA or miR), small non-coding RNAs that negatively regulate gene expression, are increasingly being characterized as important regulators of mitochondrial function in obesity (Murri and El Azzouzi, 2018). As acknowledged by Bandiera et al. (2013), miRNA can be found in mitochondria acting within the organelle, while nuclear-encoded miRNAs can act at mitochondria or in the nucleus/cytosol on genes encoding mitochondrial proteins.

In skeletal muscle of high-fat diet -fed mice, mitochondrial function is impaired (Martins et al., 2018) and miR-1 is decreased in soleus and gastrocnemius muscles (Frias et al., 2016, 2018). Thus, miR-1 expression seems to be related to mitochondrial function.

MiR-1 is the most abundant microRNA in human skeletal muscle (Liang et al., 2007) and myomiR is recognized as an important regulator of skeletal muscle development (Chen et al., 2006). Experiments in skeletal muscle satellite cells and primary myoblasts have suggested that miR-1 promotes myoblast differentiation (Chen et al., 2010). Of note, miR-1, induced during myogenesis, efficiently enters the mitochondria where it unexpectedly stimulates, rather than represses, the translation of specific mitochondrial genome-encoded transcripts (Zhang et al., 2014).

Therefore, the purpose of this study was to investigate the role of miR-1 in skeletal muscle mitochondrial function and the potential of miR-1 to treat obesity-related disorders including insulin resistance. Our findings suggest miR-1 can increase muscle oxidative metabolism and mitochondrial content in primary mouse myotubes and *in vivo* improves peripheral insulin sensitivity, providing a new insight into miR-1 function in skeletal muscle.

## Materials and Methods

### Ethics and Animals

The Experimental Animal Ethics Committees of ICB-USP and from the Institute of Biology of the University of Campinas approved the experimental procedure of this study. Male wild-type C57BL/6 J mice were obtained from the Facility for Mice Production at the Department of Pharmacology of the Institute of Biomedical Sciences (ICB) of the University of São Paulo (USP) and were maintained at 12:12-h light–dark cycle and 23°C ± 2°C. The animals were housed in cages (2–3 animals/cage) and received a standard diet (Nuvilab-Nuvital Nutrients Ltd., Parana, Brazil) and water *ad libitum* until the beginning of the experimental period.

### Experimental Design

Eight-week-old male wild-type C57BL/6J mice (*n* = 35) were randomly divided into two groups: C- fed a balanced diet (C group *n* = 12) (cat.151, Pragsoluções Biociências, Jaú, SP, Brazil) and H- fed a high-fat diet (H group, *n* = 23) (cat.10, Pragsoluções). After 6 weeks of diet, part of the animals in the H group received intramuscularly miR-1 precursor plasmid (H + mir-1a-1, *n* = 12) or negative control (scrambled control, *n* = 11), as described in the next section. Animals in the C group received a scramble control plasmid. Body weight was measured every week and intraperitoneal glucose and insulin tolerance tests (ipGTT and ipITT) were performed 1 week before euthanasia. After 28 days of the injection, animals were euthanized (between 1 and 3pm) by decapitation, and gastrocnemius (GA) and soleus muscles (SO) were carefully dissected from the surrounding tissue; GA was frozen in liquid nitrogen and stored at −80°C until analyses and SO was freshly used for insulin incubation studies. Adipose tissue depots were dissected and weighed to evaluate adiposity levels.

### *In vivo* miR-1 Therapy

*In vivo* experiments involving transfection of mouse pre-microRNA-1 expression construct (cat#MMIR-1a-1-PA-1, System biosciences) or Scramble negative control construct (cat#MMIR-000-PA-1) were conducted on GA muscle. Mice were submitted to a small incision in the skin to apply four injections (10 μl, 40U) of Hyaluronidase (Sigma #H3506, Germany). After 30 min, GA muscle received four injections (10 μL each) of the miR-1 precursor expression vector (1.25 μg/μL) in lateral and central sides of the muscle or negative control and the plasmid was introduced into muscle cells by electroporation, as described previously (Silva et al., 2019). Delivery of the plasmid was confirmed by visualization of GFP-positive fibers and DAPI, for nuclei identification, in GA sections using a fluorescence microscope Axio Scope.A1 (Carl Zeiss Microscopy GmbH, Göttingen, Germany). Contralateral non-injected GA muscle was used as a negative control.

### ipGTT, ipITT, and Serum Insulin Measurement

GTT and ITT were performed in mice who had been fasted for 6 h, as previously described (de Mendonça et al., 2020b). The plasma glucose disappearance rate (K_ITT_) during the 4- to 16-min period following the insulin injection was taken as a measure of insulin action (Bonora et al., 1989). During GTT, 20 μL of blood was also collected and homogenized with EDTA for insulin measurements. Serum insulin was measured using EZMRI- 13K kit (The Antibody Registry, 2021f).

### Cell Culture Conditions and Treatments

Myoblast C2C12 cells (ATCC #: CRL-1772, RRID:CVCL_0188) (SciCrunch, 2021) were maintained in high glucose DMEM (SIGMA, St. Louis, MO, United States) supplemented with 10% fetal bovine serum and 1% penicillin/streptomycin (10,000 UI/mL streptomycin and 10,000 UI/mL penicillin) under humidified conditions with 5% CO2 at 37°C. After total confluence was achieved, the cells were differentiated using DMEM containing horse serum (HS) 2% for 5 days. Differentiated C2C12 cells were exposed to 500 μM palmitic acid (PA) or 1% bovine serum albumin (BSA) (vehicle control) for 24 h. After treatment, the cells were washed twice with cold PBS, and total RNA was isolated using TRIzol reagent (Thermo Scientific) for analysis of miR-1 expression.

For gain-of-function experiment, undifferentiated C2C12 cells were transfected with LNA miR-1 mimic (miR-1 mimic; 472818-004 Exiqon) or scramble control (Scr; 199006-002 Exiqon), as previously described (Frias et al., 2018). Transfection media was replaced for differentiation media for 72 h and during the last 24 h, cells were treated with 500 μM PA or 1% BSA. Cells were washed with PBS and lysed in TRIzol reagent to measure mRNA expression by RT-qPCR. For quantification of p-AKT levels, the same procedure described above was done, however, after 96 h of the transfection, C2C12 cells were stimulated with 7 nM insulin for 15 min. Cells were lysed in a RIPA buffer containing protease inhibitors and western blot for p-AKT (Ser473) was performed.

### Primary Muscle Cells Transfection and Mitochondrial Oxygen Consumption

Primary mouse skeletal muscle cells were obtained, as described in Araujo et al. (2020) and plated in a 24-well seahorse plate, and differentiated using DMEM containing HS 10% for 3 days (*n* = 6). miR-1 mimic (25 nM) or Scr (25 nM) was transfected in 100 μL OptiMEM medium containing 3 μL of Lipofectamine RNAiMAX (Life Technologies) for 48 h. After 24 h of transfection, cells were treated with 500 μM PA or 1% BSA. Mitochondrial oxygen consumption was evaluated, as previously described (Lima et al., 2019), using a Seahorse analysis (XF24; Agilent Technologies Inc., Santa Clara, CA, United States). Non-mitochondrial OCR values were subtracted from all data before being used for the analyses. After each assay, cells were fixed and crystal violet was stained for cell number normalization by measuring absorbance at 590 nm. For citrate synthase activity, cells were plated in 12-well, and the same protocol described above was performed.

### Quantification of MicroRNA and mRNA Expression by RT-qPCR

Total RNA was extracted from GA muscle using miRVana Paris RNA Isolation kit (Thermo Fisher Scientific) according to the manufacturer’s instructions. Quantification of miR-1 expression was performed as described in Frias et al. (2016). MicroRNA expression was normalized to sno234 levels.

For mRNA expression, cDNA synthesis was performed using the High Capacity kit (Thermo Fisher Scientific) from 500 ng of total RNA extracted from C2C12 cells. All PCR reactions were performed using diluted (1:10) cDNA template, forward and reverse primers (200 nM each) for Pdk4, Pgc1a, Cpt1b, Acot2 genes (Frias et al., 2018), and Power SYBR Green PCR Master Mix (Thermo Fisher Scientific). For normalization of expression, the constitutive gene Hprt1 was used.

### Western Blot

The two mouse SO muscles were isolated, attached to stainless steel clips to maintain resting tension, and preincubated with Krebs-Ringer bicarbonate buffer containing glucose in the presence or absence of insulin 7 nM, as previously described (de Mendonça et al., 2020a). The muscles were briefly washed in cold KRBB at 4°C, dried on filter paper, and frozen in liquid N2. Proteins were extracted from SO and GA muscles and C2C12 frozen samples and loaded into polyacrylamide gels, separated by SDS-PAGE, and transferred to nitrocellulose membranes. Membranes were blotted with primary antibodies overnight at 4°C [p-AKT (Ser473) (The Antibody Registry, 2021e); AKT (The Antibody Registry, 2021a), AMPK (The Antibody Registry, 2021b), and p-AMPKα (Thr172) (The Antibody Registry, 2021c), Cell Signaling; GAPDH (The Antibody Registry, 2021d), Abcam] and after incubation with peroxidase-conjugated secondary antibodies for 1h at room temperature, the detection was performed by C-Digit Imager using Clarity^TM^ Western ECL (Bio-Rad).

### Citrate Synthase Activity

Citrate synthase activity was measured in primary myotube cells and in gastrocnemius muscles (10 mg) homogenized in 1:100 (wt/vol) of extraction buffer, as previously described (Alp et al., 1976). Enzyme activities were assessed in duplicate and measurements were performed every 10 s over a 3 min period on Spectramax M5 spectrophotometer (Molecular devices, Sunnyvale, CA, United States). The results were expressed on a protein basis as determined by the BCA protein assay kit (Thermo Fisher Scientific, Waltham, MA, United States).

### Statistical Analysis

Data are presented as mean ± S.D. The differences between the two groups were assessed by *t*-test and differences among three groups were assessed by one-way ANOVA, followed by Tukey’s post-test. A *p*-value of less than 0.05 was considered statistically significant.

## Results

### Intramuscular Injection of miR-1 Precursor Improves Insulin Resistance in Obese Mice

**FIGURE 1:**
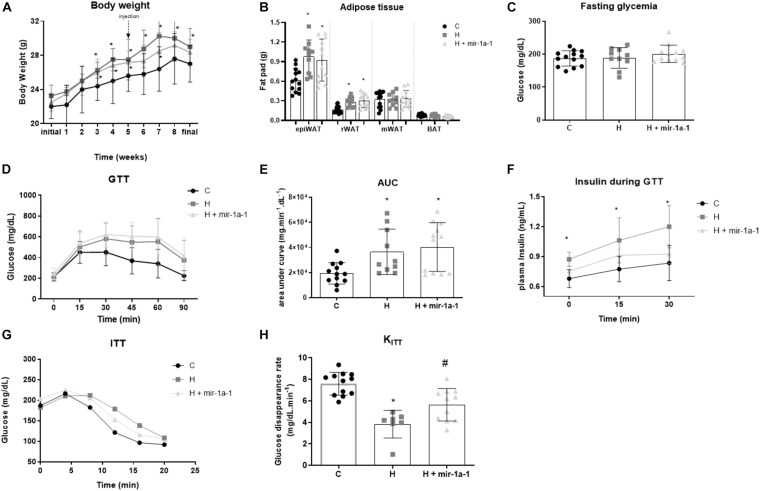
Intramuscular miR-1 precursor (mir-1a-1) replacement therapy improves insulin resistance of obese mice. **(A)** Time-course of weight gain; **(B)** white adipose tissue (WAT) mass: epi = epididymal; r = retroperitoneal, m = mesenteric and brown adipose tissue (BAT) mass; **(C)** Glycemia after a 6h-fasting; **(D)** Glucose tolerance test (GTT) curve; **(E)** Area under the curve of GTT; **(F)** Serum insulin during GTT; **(G)** Insulin tolerance test (ITT) curve; **(H)** K_ITT_ calculated from ITT curve. *#*p* < 0.05 as indicated by one-way ANOVA followed by Tukey’s post-test. (*) vs. control (C), (#) vs. obese (H); *n* = 7–12.

**FIGURE 2:**
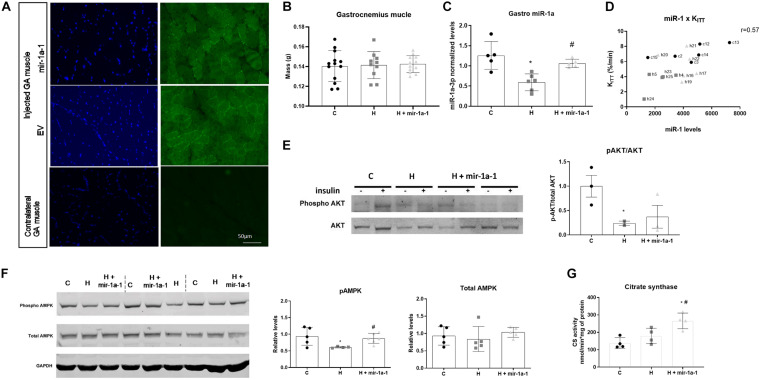
Increased miR-1 levels in gastrocnemius muscle of obese mice restores pAMPK levels. **(A)** Representative fluorescence images of GFP-positive fibers in gastrocnemius (GA) sections after 28 days of transfection of the empty vector (EV) or miR-1 precursor (mir-1a-1) and contralateral non-injected GA. Blue = DAPI for nuclei identification; Green = GFP-positive fibers; **(B)** GA mass (*n* = 10–13); **(C)** miR-1 levels in GA muscle of control (C), obese (H) and miR-1 precursor-treated obese (H + mir-1a-1) mice (*n* = 5); **(D)** correlation analysis between miR-1 expression and K_ITT_ values (*n* = 5); **(E)** Representative blots of phospho and total AKT levels after stimulation of soleus muscle with insulin. Phospho and total AKT were normalized by ponceau-stained total protein content and phospho/total AKT ratio was calculated (*n* = 2–3); **(F)** Representative phospho and total AMPK and GAPDH levels. Relative phospho and total AMPK content was calculated after normalization with GAPDH (*n* = 5); **(G)** Citrate synthase activity in gastrocnemius muscle (*n* = 4). *#*p* < 0.05 as indicated by one-way ANOVA followed by Tukey’s post-test. (*) vs. control (C), (#) vs. obese (H).

Mice were fed a high-fat diet (HFD) for 6 weeks and had increased body weight compared to control and intramuscular (i.m.) injection of miR-1 precursor (mir-1a-1), which did not affect weight gain in the following 4 weeks after injection (Figure 1A). An increment in fat mass, glucose intolerance, insulin resistance, and hyperinsulinemia were observed in obese mice after 10 weeks of high-fat diet (Figures 1B–H). Treatment with i.m. miR-1 precursor improved peripheral insulin resistance in obese mice as indicated by K_ITT_ (Figure 1H), but had no effect on fasting glycemia or glucose tolerance (Figures 1C–E).

mir-1a-1 and empty vector plasmids were effectively delivered in GA muscle as GFP-positive fibers are detected after 28 days of the injection of mir-1a-1 vector (miR-1 precursor) (Figure 2A). As expected in the muscle of the contralateral non-injected leg, there were no GPF-positive fibers (Figure 2A). We measure miR-1 levels in the GA muscle of obese and obese treated with mir-1a-1 vector and, as expected, miR-1 levels were decreased in the GA muscle of HFD-fed mice, and mir-1a-1 treatment restored miR-1 levels (Figure 2B). There was no difference in GA mass among the groups (Figure 2C). Interestingly, the levels of miR-1 in GA muscle positively correlated with K_ITT_ (Figure 2D).

We next measured phospho/total Akt ratio in insulin-stimulated soleus muscle as an indicator of insulin sensitivity in skeletal muscle. Feeding a HFD decreases the ratio of p-Akt to total Akt, however, treatment with mir-1a-1 plasmid had no effect on p-AKT levels (Figure 2E). AMPK activation in skeletal muscle has beneficial effects on glucose uptake, independent of insulin sensitivity (Koistinen et al., 2003). Thus, we analyzed the levels of AMPK in mouse GA muscle. As predicted, p-AMPK is reduced in overweight mice and i.m. injection of mir-1a-1 plasmid restored p-AMPK to C group levels (Figure 2F). AMPK activation is related to increased mitochondrial biogenesis, therefore we measured citrate synthase (CS) activity, a marker of mitochondria content and muscle oxidative capacity (Larsen et al., 2012), in GA muscle of the animals. We observed a higher CS activity in mir-1a-1-treated GA muscle compared to those from control and HFD mice (Figure 2G).

### Restoring miR-1 Expression in PA-Treated Cells Increases Expression of Genes Related to Mitochondrial Function

**FIGURE 3:**
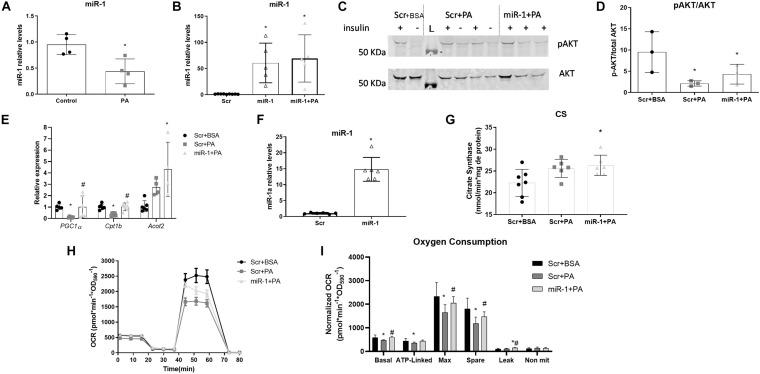
Overexpression of miR-1 protects PA-treated primary skeletal muscle cells from reducing mitochondrial oxidative capacity. **(A)** miR-1 levels in PA-treated myotubes (*n* = 4), (*p < 0.05 vs. control); **(B)** overexpression of miR-1 in PA-treated cells (*n* = 5), (*p < 0.05 vs. Scr); **(C)** Representative blots of phospho and total AKT levels after insulin treatment of control (Scr), PA-treated (Scr + PA) and miR-1 overexpressing cells treated with PA (*n* = 6), L = protein ladder; **(D)** phospho and total AKT were normalized by total protein content stained with amido black and p/total AKT ratio was calculated in insulin-stimulated cells (*n* = 3) (*p < 0.05 vs. Scr + BSA); **(E)** mRNA expression of mitochondrial and beta-oxidation markers (*n* = 6), (*#*p* < 0.05: (*) vs. Scr + BSA, (#) vs. Scr + PA); **(F)** overexpression of miR-1 in primary myotubes (*n* = 6) (*p < 0.05 vs. Scr); **(G)** Citrate synthase activity in primary myotubes (*n* = 6), (*p < 0.05 vs. Scr); **(H)** Oxygen consumption rates (OCR) corrected by non-mitochondrial OCR; **(I)** Basal, ATP-linked, proton leak (leak), spare capacity (spare) and non-mitochondrial (non-mit) OCR from primary myotubes transfected with miR-1 or scrambled control and treated with palmitic acid or vehicle for 24 h (*n* = 6). *#*p* < 0.05: (*) vs. Scr + BSA (C), (#) vs. Scr + PA.

Using an *in vitro* model of obesity we investigated the effects of miR-1 specifically in myotubes. PA-treated cells had decreased levels of miR-1 (Figure 3A) and transfection of miR-1 mimic, effectively overexpressed miR-1 in myotubes treated with PA (Figure 3B).

According to *in vivo* results, overexpression of miR-1 in C2C12 myotubes and treatment with PA could not prevent the reduction of p-Akt levels in insulin-stimulated cells (Figures 3C,D).

AMPK activation is related to increased mitochondrial fatty acid oxidation. Remarkably, in C2C12 cells overexpression of miR-1 increases p-AMPK levels and miR-1 inhibition decreases p-AMPK levels (Supplementary Figure 1). Since p-AMPK levels and higher CS activity were associated with increased miR-1 levels in GA muscle of obese animals, we measured the transcript levels of proteins involved in mitochondrial β-oxidation in C2C12 cells treated with PA and transfected with miR-1. We found *Pgc1a*, *Cpt1b*, and *Acot2* were increased in PA-treated cells overexpressing miR-1 compared to PA-treated cells (Figure 3E) suggesting that miR-1 induces a more oxidative metabolism in C2C12 myotubes.

### miR-1 Overexpression Protects PA-Treated Primary Skeletal Muscle Cells From Reducing Mitochondrial Oxidative Capacity

MiR-1 was overexpressed in primary myotubes (Figure 3F). After treatment with palmitic acid, CS activity (Figure 3G) and mitochondrial oxygen consumption, using a Seahorse analyzer (Figures 3H,I), were measured. PA-treated myotubes had no difference in CS activity compared to the control, but overexpression of miR-1 increased CS activity in PA-treated cells (Figure 3G). Non-mitochondrial respiration was calculated, and it was not different in PA-treated cells compared to control cells. Significant differences were observed in basal, ATP-linked, and maximum mitochondrial oxygen consumption rate (OCR), in primary skeletal muscle cells treated with PA compared to control cells (Figure 3I). Mitochondrial oxidative capacity, as suggested by mitochondrial reserve capacity (spare capacity), was also reduced in palmitic acid, as compared to control cells. Transfection of miR-1 mimic prevents PA in decreasing mitochondrial basal and maximal OCR and spare capacity (Figure 3I). Notably, miR-1 also increases the proton leak compared to control and PA-treated cells, suggesting miR-1 uses other mechanisms to regulate mitochondrial ATP production (Figure 3I).

## Discussion

MiR-1 is a well-known myomiR that is crucial for myotube differentiation, which is dysregulated in the skeletal muscle of obese and diabetic individuals (Chen et al., 2012; Frias et al., 2016, 2018; Dahlmans et al., 2017). However, miR-1 functions in diabetes and obesity are still underexplored. As this study describes, miR-1 may have a role in mitochondrial function in the skeletal muscle of obese subjects and intramuscular miR-1 precursor replacement therapy improves peripheral insulin resistance in obese mice.

Strategies that can improve insulin sensitivity and glycemic control are used in the management of obesity-related T2DM. Current therapy in the management of T2DM has been shown to exert beneficial effects in T2DM by modulating mitochondrial function (Pinti et al., 2019; Apostolova et al., 2020; Fiorentino et al., 2021). Mitochondrial function has been associated with muscle insulin resistance and T2D in multiple studies (Petersen et al., 2004, 2005; Ritov et al., 2005, 2010; Befroy et al., 2007; Koves et al., 2008; Phielix et al., 2008; Minet and Gaster, 2010, 2011; Fiorentino et al., 2021). Mitochondrial function has been associated with deficient oxidative capacity, resulting in increased amounts of intramyocellular lipids. Lipotoxicity interferes with insulin signaling, causing insulin resistance (Petersen et al., 2004). Impaired mitochondrial substrate oxidation has both been shown in the insulin resistant offspring of T2D patients (Befroy et al., 2007) and exercising T2D patients *in vivo* (Schrauwen and Hesselink, 2008). Thus, a reduced TCA cycle flux is both a marker and a maker of the diabetic phenotype (Gaster et al., 2012). Even though mitochondrial function has been suggested to play an important role in the pathogenesis of insulin resistance and T2DM, some studies have observed normal mitochondrial function in diet-induced obesity models (Garcia-Roves et al., 2007; Brøns et al., 2012; Stephenson et al., 2012). Here, we show that palmitic acid impairs mitochondrial respiration and possibly fatty acid oxidation in mouse myotubes. Our findings corroborate previous studies demonstrating the increase in free fatty acids metabolites inhibits ATP synthesis in isolated mitochondria from mouse and human skeletal muscle (Abdul-Ghani et al., 2008) and that saturated free fatty acids decrease both mitochondrial hyperpolarization and ATP generation in C2C12 cells and primary myotubes (Hirabara et al., 2010).

These findings show that overexpression of miR-1 in myotubes prevents abnormalities in mitochondrial function induced by the saturated fatty acid in skeletal muscle. A relationship between miR-1 and mitochondria has been established. During skeletal muscle differentiation, miR-1, a nuclear-encoded miRNA, translocates into the mitochondrial compartment to induce the expression of mitochondrial genes (Zhang et al., 2014). Besides that, after an acute bout of endurance exercise, a well-known inducer of mitochondrial biogenesis in skeletal muscle, miR-1 expression is increased in the quadriceps of mice (Safdar et al., 2009).

Low levels of miR-1 have been described in the skeletal muscle of insulin resistant mice and patients (Chen et al., 2012; Frias et al., 2016, 2018; Dahlmans et al., 2017) and, as demonstrated by our data, in cultivated insulin resistant myotubes. Interestingly, treatment with PPARα agonist, an inducer of fatty acid oxidation, decreases lipid deposition and restores miR-1 levels in soleus muscle of obese mice (Frias et al., 2018). This suggests that up-regulation of miR-1 is associated with higher oxidative metabolism. In addition, exercise training, which helps restore mitochondrial function in T2DM patients (van Tienen et al., 2012), acutely up-regulates miR-1 expression in the skeletal muscle of obese mice (Safdar et al., 2009). Consistent with these results, our data show that miR-1 increases mitochondrial biogenesis and muscle oxidative metabolism in insulin resistant myotubes.

In the present study, peripheral insulin sensitivity was increased in obese mice treated with miR-1 precursor without weight loss. Studies have shown that the capacity for AMPK-mediated glucose uptake is intact in muscle cells from patients with T2DM, while insulin-induced glucose uptake is impaired (Koistinen et al., 2003). Accordingly, AMPK was restored in the gastrocnemius muscle of miR-1 treated mice and may have contributed to insulin-independent glucose transport in skeletal muscle of miR-1 precursor treated animals. A previous report from our group demonstrated that in C2C12 cells, overexpression of miR-1 increases basal glucose uptake (Frias et al., 2018). This study showed that AMPK phosphorylation is stimulated in C2C12 cells transfected with miR-1. Moreover, activation of liver AMPK by metformin, a first-line antidiabetic drug, improves mitochondrial respiratory activity along with improved hyperglycemia in high-fat- diet-fed mice (Wang et al., 2019). Our study did not observe weight loss after miR-1 therapy. Our data corroborate previous findings that treatment of SVF cells from brown fat with LNA miR-1 inhibitor caused no effect on adipogenesis or brown fat enriched markers (Sun et al., 2011).

In conclusion, this study suggests that miR-1, through activation of AMPK, increases muscle oxidative metabolism and mitochondrial content in skeletal muscle, which in turn improves insulin sensitivity. Our data suggest a novel role of miR-1 in insulin resistance.

## Data Availability Statement

The raw data supporting the conclusions of this article will be made available by the authors, without undue reservation.

## Ethics Statement

The animal study was reviewed and approved by the Ethics Committee on Animal Use of the Biomedical Sciences Institute (University of São Paulo) (CEUA-ICB/USP).

## Author Contributions

AR conceived and designed the research. AR, AB, AS, DG, FF, GM, HA, LS, MM, and WS acquired, analyzed, and interpreted data. AR wrote the manuscript. All authors contributed to the article and approved the submitted version.

## Conflict of Interest

The authors declare that the research was conducted in the absence of any commercial or financial relationships that could be construed as a potential conflict of interest.
