# Supplementary material for: Intramuscular Injection of miR-1 Reduces Insulin Resistance in Obese Mice
Source: Front Physiol. 2021 Jul 6;12:676265. doi: 10.3389/fphys.2021.676265 (PMC8290840; doi:10.3389/fphys.2021.676265)
Supplement: Supplementary file 1 [file Table_1.DOCX]

Supplementary Material

**
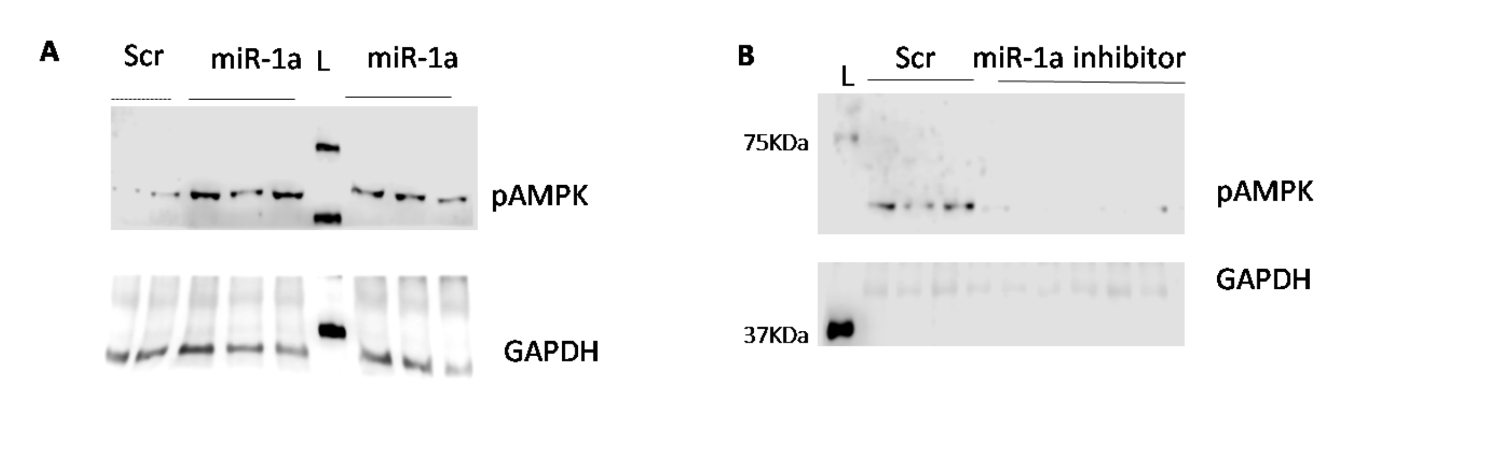
**

**Supplementary Figure 1. miR-1a controls pAMPK levels in C2C12 cells.** Blots of phospho-AMPK and GAPDH levels in C2C12 cells transfected with miR-1a mimic **(A)** or inhibitor **(B)** and scramble (Scr) control (n=6). L= protein ladder
